# Advocacy Recruiting for Huntington’s Disease Clinical Trials

**DOI:** 10.1371/currents.RRN1230

**Published:** 2011-04-12

**Authors:** LaVonne Goodman, Cherrie Sia, Roger Carnes, Louise Vetter, Fred Taubman, Charles S. Venuto, Andrew McGarry, Karl Kieburtz, Pinky Agarwal

**Affiliations:** ^*^Huntington's Disease Drug Works; ^†^Evergreen Neuroscience Institute; ^‡^Northwest Chapter Huntington's Disease Society of America; ^§^Huntington's Disease Society of America and ^#^University of Rochester Medical Center

## Abstract

Recent clinical trials for Huntington's disease (HD) have been slowed by the inability to complete enrollment in a timely manner. We report a successful advocacy-based recruiting approach at Evergreen Neuroscience Institute, a new Huntington Study Group (HSG) investigative site that lacked an HD patient base. By partnering with community advocates and utilizing web-based advocacy group alerts, Evergreen ranked third of 27 North American sites conducting the Study of ACR16 for the Treatment of Huntington's disease (HART) for number of participants, and first for rate of recruitment -- all while decreasing the time and financial resources needed for site-based recruiting. To our knowledge this is the first published outcome study for advocacy recruiting in any disease population.

## Introduction

Historically the vast majority of participants for HD clinical trials have been recruited from patient populations receiving medical care at institutions associated with the investigative site. Recruitment for CARE-HD, the North American clinical trial of coenzyme Q10 and remacemide [1] was so rapid using these methods that a lottery selection was required for the 347 participants enrolled between July 1997 and June 1998 (Karl Kieburtz, private communication March 14, 2011).  However, because there are concurrent HD trials now underway, requiring larger numbers of participants, it has been difficult to complete enrollment in a timely manner.  The interval needed to recruit 220 participants for HART was nearly two years, from October 2008 to August 2010.  Now at 3 years, the ongoing trial of high-dose coenzyme Q10 (2-CARE) [2] has recruited 509 of the 608 participant goal (Merit Cudkowicz, private communication, March 17, 2011), while the more recent trial of high-dose creatine (CREST-E) [3] has enrolled 187 of the needed 650 participants over 18 months (Stephen Hersch, private communication, March 13, 2011). To help address these recruitment needs, it is important to explore novel strategies that can augment traditional center-based approaches for HD recruiting. 

## Methods

The Evergreen recruiting effort followed an earlier project in 2009, in which community advocates had created a survey instrument that assessed levels of clinical research interest, awareness, and literacy among 100 members of 5 HD support groups in Washington State and Idaho, and measured the impact of a 1-hour education session on these variables via a second survey [4].  Support group members demonstrated both increased knowledge and increased desire for participation in clinical research. Through this project local families had exposure to advocates which fostered both familiarity and trust. We believe the positive impact of the earlier project may have had beneficial impact on the Evergreen experience.

Lacking an internal HD patient base, Evergreen relied on recruiting from both grass-roots and multiple web-based advocacy efforts.  Community advocates presented HART trial information in three small group sessions (15-30 attendees per session), that included 2 local support groups which had been involved in the prior education project, and a workshop during the 2010 HDSA Northwest Chapter symposium.  Instead of a traditional lecture presentation, the format was conversational, interactive, and encouraged questions. Specific talking points included (1) importance of clinical trials, (2) rationale for bringing the intervention to trial, (3) results and safety data from an earlier phase trial, (4) inclusion and exclusion criteria, (5) description of number of visits and procedures, and (6) Evergreen contact information.  At the conclusion of each session, Institutional Review Board (IRB)-approved printed materials were also made available.  

Concurrently the Huntington’s Disease Society of America (HDSA) [5] provided information through pamphlets delivered by U.S mail to local families on the HDSA mailing list, and through national and Northern California branch HDSA web alerts.  HDSA also notified the national HD family community of trial availability through HDTrials.org, a community-based web source for notification of clinical trials [6].  

## Results

Evergreen screened 17 individuals over an 11-month enrollment period (Table 1: demographics: gender, age, distance traveled). Though 3 failed entry criteria, 14 were enrolled for HART. Though they entered the trial late in the enrolling period, the total number of participants recruited at Evergreen was third among 27 North America recruiting centers, while the rate of recruiting at Evergreen was higher than any other center (table 2).  A third significant benefit was that minimal time and site financial resources were needed for recruiting efforts.  One of the Evergreen participants did not complete the trial, failing only the final post-drug follow-up, and was one of the 13 sites that reported single drop outs. 

**Figure:**
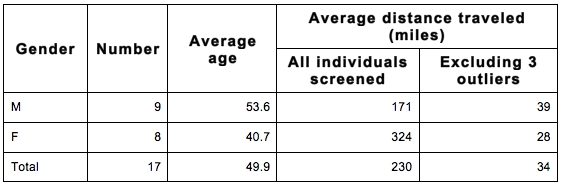
Table 1. Demographics for 17 individuals screened at Evergreen for HART. The overall average is skewed by three outliers who traveled hundreds to thousands of miles

**Figure:**
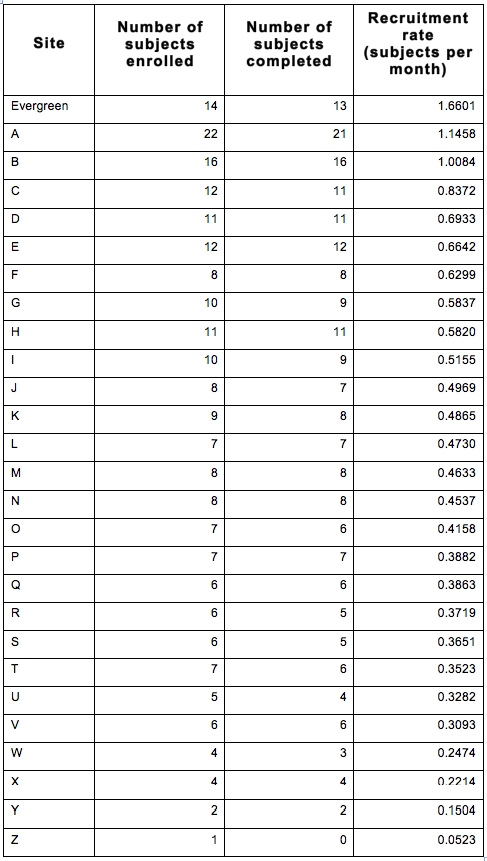
Table 2. HSG recruitment rates  rate computed as (no. subjects enrolled)/(total days open) x (30 days/month)

### Sources of referral to Evergreen 

Nine of the 17 individuals cited local advocates as the principle referral source. Five in this group volunteered for contact on the first day of open enrollment. Referral for the majority of remaining recruits came from 5 web source alerts: 2 from Northern California HDSA chapter, 1 from the national HDSA, 1 from  HDTrials.org, and 1 from Huntington's Disease Discussion (HUNT-DIS), an HD community web discussion group.  Two cited source information from the HSG web site.  Another was referred from a local neurologist.  In summary, 14 of 17 individuals coming to Evergreen received source information from grassroots and web-based advocacy efforts.  Provision of trial-specific written materials generated no recruits.  Figure 1 summarizes the sources of Evergreen referral.

**Figure:**
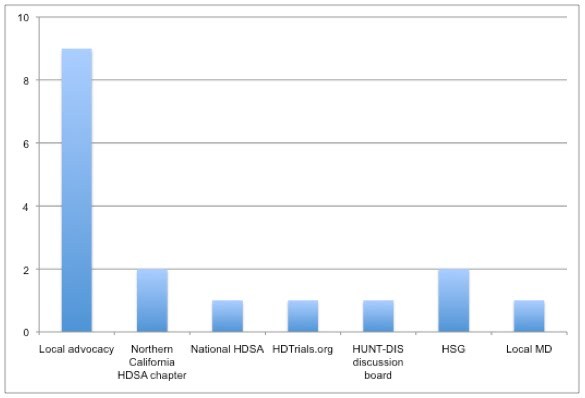
Figure 1. Sources of referral for 17 individuals screened at Evergreen for HART

### Sources of referral to other sites 

 Referral sources to Evergreen, with no internal patient base, differed from referral sources to sites with a patient base. Using data combined across centers the reported referral source was: site personnel (66%), off site neurologist (12%), self (5%), "other" specialist (4%), "other" (4%), HDSA website (2%), HSG website (2%). Table 3 summarizes HART referral sources combined across all centers except Evergreen.

**Figure:**
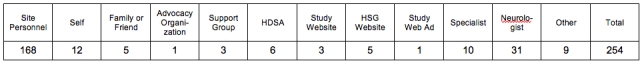
Table 3. Referral sources from all participating North American HART sites except Evergreen. Each subject may have listed more than one referral source. Referrals that were NOT cited to be a source of recruitment were newspaper article/ad, radio, television, site website, geneticist, psychiatrist, HSG call center, minority recruitment activity

## Discussion

One advocacy role of groups acting on behalf of specific disease populations has been to communicate information and stimulate interest in clinical research participation.  HDSA utilizes printed newsletters, national and local websites, teleconferences, and annual meetings at both the national and field/chapter levels.  HDSA has worked closely with HSG to provide education during annual workshops, and through local chapters and affiliates at individual investigator sites.  However, it is unclear how information from HDSA or other disease advocacy organizations translates to clinical research participation.   

The advocacy role of individuals in clinical trial recruiting is less well defined, and as discussed in a Hastings Center Review, has been quite varied [7].  Individual advocates have served as surrogate decision makers for mentally impaired individuals [8] and have assisted investigators with trial design or consenting procedures [9]. In each of these cases advocate roles are defined by parent research facilities. 

The role of individual community advocates specific to the recruiting process varies with the disease organization. Parkinson’s Disease Foundation, the North Central Cancer Group, and the Vanderbilt-Ingram Cancer Center have initiated in-person community advocate training programs to assist with recruiting efforts that range from half-day to three day intensive training sessions [10] [11] [12].  HDSA has recently implemented a Diplomat program that utilizes webinar training of volunteers and provides written materials for use at educational gatherings and support groups [13].  However, it is unclear how any of these programs define advocate roles or responsibilities, nor has there been reporting of outcomes based on these programs.

To our knowledge the Evergreen experience is the first reported outcome study of advocate recruiting for any disease.  We believe that several factors contributed to the positive outcome at the local level: (1) the establishment of partnering roles between site investigators and community advocates (2) relatively high advocate knowledge of the HD clinical research process served to validate the advocate role on both sides of the process, between site and advocate, and between advocate and local HD families, and (3) previous community familiarity and trust developed for local advocates.  

The Evergreen experience also shows the important recruiting power of web-based dissemination of information. The collaboration with HDSA was a vital contributing factor, at both the national and Northern California level.  An important contribution was made by smaller web-community sites. 

## Conclusions

Successful recruitment for HART participants at Evergreen Neuroscience Institute shows that collaborative efforts between a clinical research site and advocacy recruiting at both the community and web-based levels can increase enrollment in HD clinical trials. Advocate recruiting at the community level accounted for the larger number of Evergreen recruits and resulted in efficient early enrollment. Web-based recruiting efforts accounted for the larger number of later recruits.  Further, the degree of success at Evergreen shows that advocate based recruiting for HD can efficiently and cost-effectively deliver clinical trial participants at a level that is similar to established centers with high recruiting rates. Though the Evergreen experience shows that advocacy-based recruiting can be successful at a site lacking an internal HD patient base, it is possible that similar efforts could augment the numbers of participants in established centers, particularly those with lower recruiting rates.

## Acknowledgments

We wish to thank all HART participants and investigative center personell for their time and effort. We thank Ann Covalt for editorial assistance and Judy Roberson for Northern California advocate assistance. 

## Funding Information

No external funding was used for this project. HDSA provided postage for mailing of Northwest U.S.A. printed materials.

##  Competing interests

Authors Venuto, McGarry, and Kieburtz receive research support from a contract between the University of Rochester and NeuroSearch. The authors have declared that no other competing interests exist. 

Ethics Approval 

All written materials supplied in this project were IRB approved.

Author roles

- Dr. LaVonne Goodman. Conception, organization and primary execution of the project. Writing of the first draft and review of the manuscript.
- Dr. Roger Carnes. Assistant in execution of the project.
- Dr. Pinky Agarwal. Director of Evergreen Neuroscience Institute. Site mentor of local advocates.  HSG physician coordinator for HART. Review and critique of manuscript.
- Dr. Cherrie Sia. Evergreen HART coordinator.
- Louise Vetter and Fred Tauber. Organization and execution of HDSA web and local mailing efforts.
- Dr. Karl Kieburtz, Dr. Charles Venuto, Dr. Andrew McGarry supplied comparison HART recruiting data.

Correspondence should be addressed to LaVonne Goodman: lavonne@hddrugworks.org 
